# Multidrug-resistant organism bloodstream infection and hospital acquisition among inpatients in three tertiary Greek hospitals during the COVID-19 era

**DOI:** 10.1007/s10096-024-04806-x

**Published:** 2024-03-26

**Authors:** Polyxeni Karakosta, Sophia Vourli, Elisavet Kousouli, Georgios Meletis, Areti Tychala, Christina Louka, Alexandra Vasilakopoulou, Efthymia Protonotariou, Vasiliki Mamali, Olympia Zarkotou, Lemonia Skoura, Spyros Pournaras

**Affiliations:** 1 Laboratory of Clinical Microbiology, Medical School, Attikon University Hospital, National and Kapodistrian University of Athens, Athens, Greece; 2 Department of Clinical Microbiology, Tzaneio General Hospital of Piraeus, Athens, Greece; 3 Department of Microbiology, School of Medicine, AHEPA University Hospital, Aristotle University of Thessaloniki, Thessaloniki, Greece

**Keywords:** Healthcare-associated infections, Multidrug resistance, Surveillance, Greece

## Abstract

From 2019 (pre-COVID-19) to 2022 (COVID-19 years), three tertiary Greek hospitals monitored MDRO bloodstream infection (BSI) and hospital acquisition relying on laboratory data. Surveillance covered carbapenem-resistant *Enterobacterales* (CRE), *Acinetobacter baumannii* (CRAB), *Pseudomonas aeruginosa* (CRPA), vancomycin-resistant enterococci (VRE), and methicillin-resistant *Staphylococcus aureus* (MRSA), in intensive care units (ICUs) and non-ICUs. Non-ICUs experienced significant increases in CRE, CRAB and VRE during the pandemic. In ICUs, CRE increased in 2021, CRAB in 2020 and 2021, and VRE in 2021 and 2022. KPC predominated among CRE. MDRO BSI and hospital acquisition incidence rates increased, driven by CRE and CRAB.

**Supplementary Information:**

The online version contains supplementary material available at 10.1007/s10096-024-04806-x.

## Introduction

The global health systems and antimicrobial resistance (AMR) were significantly affected by the COVID-19 pandemic [1] with AMR contributing to 4,95 million deaths in 2019, of which 1,27 million were directly linked to drug resistance [2]. Combating AMR requires a multifaceted approach [3] but COVID-19 has hindered progress [4, 5]. Various factors, including antibiotic use, infection prevention and control, public health policy making, and cross-border spread, influence AMR either positively or negatively [5]. While the impact of COVID-19 on AMR is not fully understood yet, ongoing surveillance is crucial to monitor trends during the pandemic. In Greece, healthcare-acquired infections affect approximately 121,000 patients annually, with a prevalence 50% higher than the European average [6, 7], often due to multidrug-resistant organisms (MDRO) [8]. Since the late 2000s, Greece has been facing an endemic situation in hospitals, specifically carbapenem-resistant Gram-negative bacilli, and the country’s AMR rates rank among the highest in Europe [9]. This study aimed to determine rates of hospital-onset MDRO bloodstream Infection (BSI) and overall Infection/Colonization among inpatients of three tertiary Greek hospitals, providing insights into increased rates during the COVID-19 years and supporting efforts to reduce MDRO rates and enhance patient care quality.

## Methods

### Study design

This 4-year study, from January 2019 to December 2022, involved three tertiary Greek hospitals: Attikon University Hospital of Athens (no of beds: 730), AHEPA University Hospital of Thessaloniki (no of beds: 680) and Tzaneio General Hospital of Piraeus (no of beds: 454). The first two are major teaching hospitals with medical and postgraduate student programs, while the third is a tertiary hospital offering residency and/or fellowship training programs. All handle complex cases in internal medicine, surgery, and pediatrics, and have intensive care units (ICUs).

Starting from January 2019, we monitored Multidrug-Resistant Organisms (MDROs), following the CDC protocol [10]. The surveillance protocol relied on culture results of clinical samples that were collected from hospitalized adult and pediatric patients. Active surveillance samples were not included and clinical evaluation of cases was not considered. Monitored MDROs included carbapenem-resistant *Enterobacterales* (CRE), carbapenem-resistant *Acinetobacter baumannii* (CRAB), carbapenem-resistant *Pseudomonas aeruginosa* (CRPA), vancomycin-resistant enterococci (VRE), and methicillin-resistant *Staphylococcus aureus* (MRSA).

All MDRO isolates from any specimen were examined to identify Laboratory-Identified (LabID) Events monthly and by location group (ICU, non-ICU). First MDRO isolates per patient and month were reported as LabID events regardless of specimen source; if a duplicate MDRO isolate was from blood, a LabID event was reported only if it represented a unique blood source (no prior MDRO isolation in the blood from the same patient within 14 days, even across calendar months]. Each LabID event was reported individually.

Surveillance was conducted prospectively with local investigators trained in the CDC protocol. Total patient days obtained from hospital records monthly served as denominator data.

### Microbiological methods

Species were identified by MALDI-ToF (Microflex LT system; Bruker Daltonics, Italy) and antimicrobial susceptibility was tested by BD Phoenix 50 (Becton Dickinson Diagnostic Systems, Hunt Valley, MD) or VITEK 2 (bioMérieux, Marcy l’Etoile, France) system. MDROs were defined by CDC protocol [10] and expanded to accommodate Greece’s endemic situation: (i) CRE: *Escherichia coli*, *Klebsiella oxytoca*, *Klebsiella pneumoniae*, *Klebsiella aerogenes* or *Enterobacter* spp. resistant to imipenem and meropenem, (ii) CRAB: *Acinetobacter baumannii* resistant to imipenem and meropenem, (iii) CRPA: *Pseudomonas aeruginosa* resistant to imipenem and meropenem, (iv) VRE: *Enterococcus faecalis* or *Enterococcus faecium* resistant to vancomycin, and (v) MRSA: *Staphylococcus aureus* resistant to oxacillin/cefoxitin. CRE isolates with elevated MICs for imipenem and meropenem underwent NG-Test CARBA 5 immunochromatographic assay to detect the five most common carbapenemases (KPC, IMP, NDM, VIM, and OXA-48) rapidly and accurately. Breakpoints were interpreted following the European Committee on Antimicrobial Susceptibility Testing guidelines [11].

### MDRO data analysis

LabID Events were classified as Community-Onset (CO) if the specimen was collected within the first 3 days after hospital admission, or Hospital-Onset (HO) if collected more than 3 days after admission.

For each MDRO, location group, hospital and year we reported:

i. Measures for MDRO Bloodstream Infection:

- HO unique blood source LabID events (from blood specimens only).
- MDRO BSI Incidence Density Rate (IDR): number of unique blood source HO LabID events, divided by patient days for the location group x 1,000.

ii. Measures for MDRO Healthcare Acquisition:

- first new HO LabID events per patient (from any specimen, no prior evidence of any infection or colonization for that specific MDRO from a previous LabID Event).
- Overall MDRO Infection/Colonization IDR: number of first new HO LabID events per patient, divided by patient days for the location group x 1,000.

Pooled MDRO IDR was presented for each location group and year with monthly and run charts for CRE and CRAB bloodstream Infection IDR. Data were categorized into pre-COVID-19 (2019) and COVID-19 (2020–2022) to assess the pandemic’s impact on MDRO incidence. Changes in IDR from pre-COVID-19 to COVID-19 years were analyzed and the ratio of these two rates (incidence rate ratio) with its 95% Confidence Interval and associated P-value [12, 13] was calculated to determine the impact [14].

To examine the prevalence of the most common carbapenemase genes across different years, we used the study population of first new HO CRE LabID Events.

## Results and discussion

Table 1 presents the pooled patient days, annual MDRO BSI and annual overall Infection/Colonization IDR for all location groups during the surveillance period. Compared to 2019, pooled patient days decreased in non-ICU but increased in ICU. The annual MDRO rates for each hospital are presented in Supplementary Table 1.

**Table 1:** Pooled multidrug-resistant organism incidence density rates by year and pathogen and Incidence rate ratios following the COVID-19 pandemic in three tertiary Greek hospitals, 2019–2022

				Overall Infection / Colonization						Bloodstream Infection					
	Pooled patient days		MDRO	Pooled first new LabID events per patient HO (n)		Pooled overall Infection / Colonization IDR (/1,000 patient days)		Incidence rate ratio (95% CI)		Pooled unique blood source LabID events HO (n)		Pooled bloodstream Infection IDR (/1,000 patient days)		Incidence rate ratio (95% CI)	
	ICU	Non-ICU		ICU	Non-ICU	ICU	Non-ICU	ICU	Non-ICU	ICU	Non-ICU	ICU	Non-ICU	ICU	Non-ICU
2019	16,048	416,834	CRE	110	170	6.85	0.41	*reference*	*reference*	47	44	2.93	0.11	*reference*	*reference*
			CRAB	190	220	11.84	0.53	*reference*	*reference*	68	50	4.24	0.12	*reference*	*reference*
			CRPSE	45	77	2.80	0.18	*reference*	*reference*	14	18	0.87	0.04	*reference*	*reference*
			VRE	15	96	0.93	0.23	*reference*	*reference*	7	14	0.44	0.03	*reference*	*reference*
			MRSA	8	90	0.50	0.22	*reference*	*reference*	6	33	0.37	0.08	*reference*	*reference*
2020	18,150	343,664	CRE	129	178	7.11	0.52	1.04 (0.80, 1.35)	**1.27 (1.02, 1.58)**	70	77	3.86	0.23	1.32 (0.90, 1.95)	**2.12 (1.45, 3.15)**
			CRAB	286	242	15.76	0.70	**1.33 (1.10, 1.61)**	**1.33 (1.10, 1.61)**	121	79	6.67	0.23	**1.57 (1.16, 2.15)**	**1.91 (1.33, 2.79)**
			CRPSE	38	65	2.09	0.19	0.75 (0.47, 1.18)	1.02 (0.72, 1.44)	10	21	0.55	0.06	0.63 (0.25, 1.53)	1.42 (0.72, 2.82)
			VRE	28	118	1.54	0.34	1.65 (0.85, 3.32)	**1.49 (1.12, 1.97)**	17	28	0.94	0.08	2.15 (0.85, 6.12)	**2.43 (1.24, 4.99)**
			MRSA	4	74	0.22	0.22	0.44 (0.10, 1.65)	0.99 (0.72, 1.37)	2	33	0.11	0.10	0.30 (0.03, 1.65)	1.21 (0.73, 2.03)
2021	27,489	383,133	CRE	306	306	11.13	0.80	**1.62 (1.30, 2.04)**	**1.96 (1.62, 2.38)**	139	92	5.06	0.25	**1.73 (1.23, 2.46)**	**2.27 (1.57, 3.33)**
			CRAB	613	358	22.30	0.93	**1.88 (1.60, 2.23)**	**1.77 (1.49, 2.10)**	282	129	10.26	0.35	**2.42 (1.85, 3.20)**	**2.81 (2.01, 3.97)**
			CRPSE	79	88	2.87	0.23	1.02 (0.70, 1.51)	1.24 (0.90, 1.71)	22	22	0.80	0.06	0.92 (0.45, 1.94)	1.33 (0.68, 2.63)
			VRE	66	171	2.40	0.45	**2.57 (1.45, 4.85)**	**1.94 (1.50, 2.51)**	43	62	1.56	0.17	**3.59 (1.60, 9.45)**	**4.81 (2.67, 9.32)**
			MRSA	10	53	0.36	0.14	0.73 (0.26, 2.13)	**0.64 (0.45, 0.91)**	3	32	0.11	0.09	0.29 (0.05, 1.37)	1.05 (0.63, 1.77)
2022	29,009	403,897	CRE	241	337	8.31	0.83	1.21 (0.96, 1.53)	**2.05 (1.70, 2.48)**	103	115	3.55	0.29	1.21 (0.85, 1.75)	**2.70 (1.89, 3.91)**
			CRAB	376	433	12.96	1.07	1.10 (0.92, 1.31)	**2.03 (1.72, 2.40)**	150	165	5.17	0.42	1.22 (0.91, 1.65)	**3.41 (2.47, 4.77)**
			CRPSE	86	141	2.96	0.35	1.06 (0.73, 1.55)	**1.89 (1.42, 2.53)**	18	50	0.62	0.13	0.71 (0.33, 1.55)	**2.87 (1.64, 5.22)**
			VRE	50	225	1.72	0.56	**1.84 (1.02, 3.53)**	**2.42 (1.90, 3.10)**	30	63	1.03	1.07	**2.37 (1.02, 6.39)**	**4.64 (2.58, 8.98)**
			MRSA	9	78	0.31	0.19	0.62 (0.21, 1.85)	0.89 (0.65, 1.22)	4	42	0.14	0.11	0.37 (0.08, 1.56)	1.31 (0.81, 2.14)

*Notes* CI: confidence interval; CRAB: carbapenem-resistant *Acinetobacter baumannii*; CRE: carbapenem-resistant Enterobacterales; CRPA: carbapenem-resistant *Pseudomonas aeruginosa*; HO: Hospital onset; ICU: Internal care unit; IDR: Incidence Density Rate; LabID: Laboratory-Identified; MRSA: methicillin-resistant *Staphylococcus aureus*; VRE: vancomycin-resistant enterococci

Bold font indicates statistical significance

Compared to 2019, CRE BSI IDR increased significantly in the wards during all COVID-19 years (112% in 2020, 127% in 2021, and 170% in 2022), while in ICUs the increase was significant only in 2021 (73%). Supplementary Fig. 1 shows a run chart tracking monthly changes in CRE and CRAB BSI IDR from 2019 to 2022. Overall CRE Infection/Colonization IDR aligned consistently with BSI IDR in both location groups. Previous reports showed varied CRE trends [15–17] with risk factors including prolonged hospital stays, invasive devices, carbapenems and corticosteroids exposure, and ICU admission [18, 19]. Inappropriate use of antibiotics in COVID-19 patients and poor adherence to infection prevention and control (IPC) practices [5] contribute to CRE emergence and transmission [20]. Interestingly, we observed that the distribution of carbapenemases has changed over time during the COVID-19 era. Among CRE isolates, KPC was the most common carbapenemase (60.9% in 2019, 66.7% in 2022) (Table 2). Metallo-β-lactamase (MBL)-producing isolates decreased from 30.0 to 18.4%, while KPC plus MBL increased (from 9.1 to 13.5%). Despite KPC and NDM being recognized as predominant carbapenemases in COVID-19 patients [21], few studies have assessed longitudinal trends in carbapenemase prevalence during the COVID-19 period, which is crucial for effective treatment strategies and infection control.

**Table 2:** Carbapenemases/β-lactamases in the group of hospital onset carbapenem-resistant Enterobacterales Laboratory-Identified Events by year in three tertiary Greek hospitals, 2019–2022

	2019		2020		2021		2022	
	N	(%)	N	(%)	N	(%)	N	(%)
Class A *(*KPC)	140	60.9	157	59.5	286	58.3	322	66. 7
Class B (Metallo-β-lactamase)	69	30.0	84	31.8	155	31.6	89	18.4
NDM	17		16		42		60	
VIM	22		34		17		11	
IMP	0		0		0		0	
phenotypic Class B^a^	30		34		96		18	
Class D (OXA-48)	0	0.0	0	0.0	2	0.4	2	0.4
Class A & Class B	21	9.1	22	8.3	47	9.6	65	13.5
KPC & NDM	0		0		3		20	
KPC & VIM	19		10		37		32	
KPC & phenotypic Class B^a^	2		12		7		13	
Class B & Class D (NDM & OXA-48)	0	0.0	1	0.4	1	0.2	3	0.6
**Total**	**230**	**100.0**	**264**	**100.0**	**491**	**100.0**	**483**	**100.0**

^a^cases where NG-Test CARBA 5 immunochromatographic assay not performed and carbapenemase production was shown by synergy between discs of meropenem and EDTA

In the ICU, the CRAB BSI IDR increased significantly (142%) from 4.24 infections/1,000 patient days (pd) in 2019 to 10.26 infections/1,000 pd in 2021 and declined to 5.17 infections/1,000 pd in 2022. Non-ICU faced very significant increases in CRAB BSI IDR, which was 241% higher in 2022 than 2019. CRAB Infection/Colonization IDR paralleled BSI IDR. The global rise in CRAB during the pandemic, reported as increased incidence or prevalence is concerning [16]. *A. baumannii* is commonly isolated among COVID-19 patients and survives for long periods on surfaces and under dry conditions [22], making prioritized preventive measures crucial. Challenges during the pandemic may compromise these measures, contributing to increased CRAB transmission. Overuse of carbapenems among hospitalized COVID-19 patients, changes in compliance to IPC programs and practices (difficulties in adhering to standard IPC precautions, inappropriate glove use, low health care workers-to-patient ratios, temporary discontinuation of conventional IPC efforts) (5) and other risk factors (prolonged hospitalization, immunosuppressive therapy, invasive devices, ICU admission), could also contribute to this surge in CRAB infections [23].

A significant increase (187%) in CRPA BSI IDR was observed in non-ICU during 2022 compared with 2019 (from 0.04 to 0.13 infections/1,000 pd). The overall Infection/Colonization IDR followed similar trend. In our study, CRPA BSI remained stable, except for a notable increase in 2022 in the wards. Literature on the pandemic’s impact on CRPA shows conflicting results, with some studies reporting a decrease and others an increase [16, 17].

In both ICU and non-ICU settings, VRE BSI IDR in non-ICU increased from 0.03 in 2019 to 0.17 and 1.07 infections/1,000 pd in 2021 and 2022, respectively. In ICU, the IDR rose from 0.44 infections/1,000 pd in 2019 to 1.56 in 2021 and 1.03 in 2022, making a substantial rise of almost 260% in ICU and 380% in non-ICU in 2021. Notably, the COVID-19 pandemic significantly increased VRE BSI and overall infection/colonization in 2021 and 2022, consistently with other studies [16, 17]. Disruption of certain infection control measures (targeted screening, isolation measures, cleaning and disinfection) [24] along with inappropriate use of antibiotics, such as vancomycin and third-generation cephalosporins, may contribute to this rise [25, 26]. Notably, a few studies reported a reduction in VRE incidence, possibly due to improved hygiene practices [16].

Between 2019 and 2022, overall MRSA BSI IDR remained stable in both ICU and non-ICU. For non-ICU, we observed a decrease of 36% in the IDR of MRSA overall infection/colonization in non-ICU settings during 2021 (Table 2). However, more than 50% of studies reported increased MRSA prevalence/incidence during the COVID-19 pandemic [16, 17] showing geographic disparities and variations. Despite the evidence from a meta-analysis linking antibiotic exposure to MRSA [27], Greece saw higher systemic antibacterial use in 2021 compared to 2019 [28]. Pre-pandemic guidelines on early detection and isolation of MRSA positive patients were proposed [29], but compliance appears unchanged, with no unavailable data. Various complex factors are likely to influence MRSA rates.

The current study has both strengths and limitations. This study is the first to establish rates for hospital-onset MDRO BSI and overall Infection/Colonization among inpatients in Greek hospitals providing a perspective on the increased rates observed during the COVID-19 years. Also, it stands out as one of the few studies that specifically examine MDRO rates during the COVID-19 era in two distinct settings: ICU and non-ICU. Additionally, the data from three hospitals, located in the three largest Greek cities (Athens, Piraeus, and Thessaloniki) were merged using the same validated methodology from the CDC, enabling reliable conclusions. However, variability between settings underscores the need for continuous monitoring within each hospital. While data covers geographically distinct regions, a lack of a representative sample hinders national benchmarking. Furthermore, the focus on inpatients limits generalizability to the community. Lastly, despite the absence of clinical evaluation of the patient to distinguish between infection and colonization, the observed trends closely parallel those seen in BSIs suggesting a common trend between these two categories.

In conclusion, compared to 2019, non-ICU settings experienced significant increases in CRE, CRAB, and VRE throughout all COVID-19 years, with CRPA increasing only in 2022 and MRSA decreasing in 2021. In ICUs, CRE increased only in 2021, CRAB in 2020 and 2021, and VRE in both 2021 and 2022. KPC was the predominant carbapenemase among CRE in all years, while MBL decreased, and CRE with both KPC and MBL increased in 2022. The COVID-19 pandemic has led to increased MDRO rates due to disruptions in infection control, reduced contact precautions and surveillance programs, challenges in isolating infected patients and increased antibiotic use among COVID-19 patients. Continuous surveillance, infection control measures and antimicrobial stewardship interventions are recommended to reduce further spread of MDROs within healthcare settings.

### Electronic supplementary material

Below is the link to the electronic supplementary material.

Supplementary Material 1

Supplementary Material 2

Supplementary Material 3

## Data Availability

The authors confirm that the data supporting the findings of this study are available within the article and its supplementary materials.

## References

[1] CDC. COVID-19: U.S. Impact on Antimicrobial Resistance, Special Report 2022. Atlanta, GA: U.S. Department of Health and Human Services [Internet] (2022) 14;399:629–55. 10.15620/cdc:117915

[2] Murray CJ, Ikuta KS, Sharara F, Swetschinski L, Robles Aguilar G, Gray A et al Global burden of bacterial antimicrobial resistance in 2019: a systematic analysis. Lancet 2022 12;399:629–655. 10.1016/S0140-6736(21)02724-0 10.1016/S0140-6736(21)02724-0 PMC8841637 35065702

[3] Executive Summary Policy Pathways to Combat the Global Crisis of Antimicrobial Resistance www.asm.org/advocacy 1 [Internet]. doi: www.asm.org/advocacy

[4] EClinicalMedicine Antimicrobial resistance: a top ten global public health threat. EClinicalMedicine 2021 1;41. 10.1016/j.eclinm.2021.101221 10.1016/j.eclinm.2021.101221 PMC8633964 34877512

[5] Monnet DL, Harbarth S Will coronavirus disease (COVID-19) have an impact on antimicrobial resistance? Euro 2020 1;25:1–6. 10.2807/1560-7917.ES.2020.25.45.2001886 10.2807/1560-7917.ES.2020.25.45.2001886 PMC7667630 33183403

[6] Kritsotakis EI, Kontopidou F, Astrinaki E, Roumbelaki M, Ioannidou E, Gikas A Prevalence, incidence burden, and clinical impact of healthcare-associated infections and antimicrobial resistance: a national prevalent cohort study in acute care hospitals in Greece. Infect Drug Resist 2017 10;10:317–328. 10.2147/IDR.S147459 10.2147/IDR.S147459 PMC5644569 29066921

[7] Ioannou P, Astrinaki E, Vitsaxaki E, Bolikas E, Christofaki D, Salvaraki A et al A Point Prevalence Survey of Healthcare-Associated Infections and Antimicrobial Use in Public Acute Care Hospitals in Crete, Greece. Antibiotics 2022 1;11. 10.3390/antibiotics11091258 10.3390/antibiotics11091258 PMC9495163 36140037

[8] Cassini A, Högberg LD, Plachouras D, Quattrocchi A, Hoxha A, Simonsen GS et al Attributable deaths and disability-adjusted life-years caused by infections with antibiotic-resistant bacteria in the EU and the European Economic Area in 2015: a population-level modelling analysis. Lancet Infect Dis 2019 1;19:56–66. 10.1016/S1473-3099(18)30605-4 10.1016/S1473-3099(18)30605-4 PMC6300481 30409683

[9] Polemis M, Mandilara G, Pappa O, Argyropoulou A, Perivolioti E, Koudoumnakis N et al COVID-19 and antimicrobial resistance: data from the greek electronic system for the surveillance of antimicrobial resistance—WHONET-Greece (January 2018–March 2021). Life 2021 1;11. 10.3390/life11100996 10.3390/life11100996 PMC8538738 34685368

[10] CDC’s National Healthcare Safety Network Multidrug-Resistant Organism & Clostridioides difficile Infection (MDRO/CDI) Module [Internet]. January 2024 2024;1–52. doi: https://www.cdc.gov/nhsn/psc/cdiff/index.html

[11] EUCAST Clinical breakpoints - breakpoints and guidance [Internet]. v 13.1 2022;15. doi: https://www.eucast.org/fileadmin/src/media/PDFs/EUCAST_files/Breakpoint_tables/v_12.0_Breakpoint_Tables.pdf

[12] H H. Analysis of Crude Data. In: Modern epidemiology, ed Rothman KJ. Boston: Little: Brown & Co; (1886)

[13] Sahai HKA (1996). Statistics in epidemiology: methods, techniques, and applications.

[14] MedCalc Software Ltd Confidence interval for a rate [Internet]. Version 22.018 2023 6;doi: https://www.medcalc.org/calc/rate_ci.php

[15] Bolikas E, Astrinaki E, Panagiotaki E, Vitsaxaki E, Saplamidou S, Drositis I et al Impact of SARS-CoV-2 preventive measures against Healthcare-Associated infections from antibiotic-resistant ESKAPEE pathogens: a Two-Center, Natural Quasi-experimental Study in Greece. Antibiot 2023 22;12:1088. 10.3390/antibiotics12071088 10.3390/antibiotics12071088 PMC10376605 37508184

[16] Abubakar U, Al-Anazi M, alanazi Z, Rodríguez-Baño J Impact of COVID-19 pandemic on multidrug resistant gram positive and gram negative pathogens: a systematic review. J Infect Public Health 2023 1;16:320–331. 10.1016/j.jiph.2022.12.022 10.1016/j.jiph.2022.12.022 PMC9804969 36657243

[17] Micheli G, Sangiorgi F, Catania F, Chiuchiarelli M, Frondizi F, Taddei E et al The Hidden Cost of COVID-19: Focus on Antimicrobial Resistance in Bloodstream Infections. Microorganisms 2023 1;11. 10.3390/microorganisms11051299 10.3390/microorganisms11051299 PMC10222833 37317274

[18] Vlad ND, Cernat RC, Carp S, Mitan R, Dumitru A, Nemet C et al Predictors of carbapenem-resistant Enterobacteriaceae (CRE) strains in patients with COVID-19 in the ICU ward: a retrospective case–control study. Journal of International Medical Research 2022 1;50. 10.1177/03000605221129154 10.1177/03000605221129154 PMC9583214 36259133

[19] Liu P, Li X, Luo M, Xu X, Su K, Chen S et al Risk factors for Carbapenem-resistant Klebsiella pneumoniae infection: a Meta-analysis. Microb Drug Resist 2018 1;24:190–198. 10.1089/mdr.2017.0061 10.1089/mdr.2017.0061 PMC5873294 28749714

[20] Langford BJ, So M, Raybardhan S, Leung V, Soucy JPR, Westwood D et al Antibiotic prescribing in patients with COVID-19: rapid review and meta-analysis. Clin Microbiol Infect 2021 1;27:520–531. 10.1016/j.cmi.2020.12.018 10.1016/j.cmi.2020.12.018 PMC7785281 33418017

[21] Ayoub Moubareck C, Hammoudi Halat D (2022). The collateral effects of COVID-19 pandemic on the Status of Carbapenemase-Producing pathogens. Front Cell Infect Microbiol.

[22] Rangel K, Chagas TPG, De-Simone SG (2021) Acinetobacter baumannii infections in times of COVID-19 pandemic. Pathogens 10. 10.3390/pathogens10081006 10.3390/pathogens10081006 PMC8399974 34451470

[23] Zhou H, Yao Y, Zhu B, Ren D, Yang Q, Fu Y et al (2019) Risk factors for acquisition and mortality of multidrug-resistant Acinetobacter baumannii bacteremia; a retrospective study from a Chinese hospital. Med (United States) 98. 10.1097/MD.0000000000014937 10.1097/MD.0000000000014937 PMC6456023 30921191

[24] van Dijk MD, Voor in ’t holt AF, Alp E, Hell M, Petrosillo N, Presterl E et al (2022) Infection prevention and control policies in hospitals and prevalence of highly resistant microorganisms: an international comparative study. Antimicrob Resist Infect Control. ;11:1–11. 10.1186/s13756-022-01165-0 10.1186/s13756-022-01165-0 PMC9727845 36474304

[25] Harbarth S, Cosgrove S, Carmeli Y (2002). Effects of antibiotics on nosocomial epidemiology of Vancomycin-resistant enterococci. Antimicrob Agents Chemother.

[26] Fridkin SK, Edwards JR, Courval JM, Hill H, Tenover FC, Lawton R (2001). The Effect of Vancomycin and Third-Generation cephalosporins on Prevalence of Vancomycin-resistant Enterococci in 126 U.S. adult intensive care units. Ann Intern Med [Internet].

[27] Tacconelli E, De angelis G, Cataldo MA, Pozzi E, Cauda R (2008). Does antibiotic exposure increase the risk of methicillin-resistant Staphylococcus aureus (MRSA) isolation? A systematic review and meta-analysis. J Antimicrob Chemother.

[28] European Centre for Disease Prevention and Control (2022) Antimicrobial consumption in the EU/EEA (ESAC-Net) - Annual Epidemiological Report 2021. Stockholm

[29] Kramer A, Wagenvoort H, Ahrén C, Daniels-Haardt I, Hartemann P, Kobayashi H et al (2010) Epidemiology of MRSA and current strategies in Europe and Japan. GMS Krankenhhyg Interdiszip [Internet] 5:Doc01. 10.3205/dgkh000144 10.3205/dgkh000144 PMC2831258 20204100

