# Supplementary material for: Multidrug-resistant organism bloodstream infection and hospital acquisition among inpatients in three tertiary Greek hospitals during the COVID-19 era
Source: Eur J Clin Microbiol Infect Dis. 2024 Mar 26;43(6):1241–6. doi: 10.1007/s10096-024-04806-x (PMC11178613; doi:10.1007/s10096-024-04806-x)
Supplement: Supplementary file 2 — Supplementary Material 2 [file 10096_2024_4806_MOESM2_ESM.docx]

**Supplementary Table 1: Descriptive data and Multidrug-Resistant Organism Incidence Density Rates per location and year in three tertiary Greek hospitals, 2019-2022**

|  | | | | | Overall Infection / Colonization | | | | | Bloodstream Infection | | | |
| --- | --- | --- | --- | --- | --- | --- | --- | --- | --- | --- | --- | --- | --- |
|  |  | Patient days | | MDRO | First LabID events per patient HO (n) | | | Overall Infection / Colonization IDR  (/1,000 patient days) | | Unique blood source LabID events HO (n) | | Bloodstream Infection IDR  (/1,000 patient days) | |
|  |  | ICU | Non-ICU |  | ICU | Non-ICU | ICU | | Non-ICU | ICU | Non-ICU | ICU | Non-ICU |
| 2019 | Attikon University Hospital | 6,553 | 192,133 | CRE | 32 | 62 | 4.88 | | 0.32 | 12 | 15 | 1.83 | 0.08 |
|  |  |  |  | CRAB | 82 | 74 | 12.51 | | 0.39 | 19 | 18 | 2.90 | 0.09 |
|  |  |  |  | CRPSE | 19 | 29 | 2.90 | | 0.15 | 4 | 8 | 0.61 | 0.04 |
|  |  |  |  | VRE | 8 | 62 | 1.22 | | 0.32 | 6 | 8 | 0.92 | 0.04 |
|  |  |  |  | MRSA | 4 | 46 | 0.61 | | 0.24 | 3 | 17 | 0.46 | 0.09 |
|  | AHEPA University Hospital | 5,570 | 141,078 | CRE | 23 | 85 | 4.13 | | 0.60 | 7 | 23 | 1.26 | 0.16 |
|  |  |  |  | CRAB | 48 | 126 | 8.62 | | 0.89 | 14 | 24 | 2.51 | 0.17 |
|  |  |  |  | CRPSE | 15 | 35 | 2.69 | | 0.25 | 7 | 9 | 1.26 | 0.06 |
|  |  |  |  | VRE | 0 | 12 | 0.00 | | 0.09 | 0 | 4 | 0.00 | 0.03 |
|  |  |  |  | MRSA | 2 | 28 | 0.36 | | 0.20 | 1 | 10 | 0.18 | 0.07 |
|  | Tzaneio General Hospital | 3,925 | 83,623 | CRE | 55 | 23 | 14.01 | | 0.28 | 28 | 6 | 7.13 | 0.07 |
|  |  |  |  | CRAB | 60 | 20 | 15.29 | | 0.24 | 35 | 8 | 8.92 | 0.10 |
|  |  |  |  | CRPSE | 11 | 13 | 2.80 | | 0.16 | 3 | 1 | 0.76 | 0.01 |
|  |  |  |  | VRE | 7 | 22 | 1.78 | | 0.26 | 1 | 2 | 0.25 | 0.02 |
|  |  |  |  | MRSA | 2 | 16 | 0.51 | | 0.19 | 2 | 6 | 0.51 | 0.07 |
| 2020 | Attikon University Hospital | 6,250 | 164,761 | CRE | 24 | 75 | 3.84 | | 0.46 | 8 | 22 | 1.28 | 0.13 |
|  |  |  |  | CRAB | 122 | 98 | 19.52 | | 0.59 | 35 | 27 | 5.60 | 0.16 |
|  |  |  |  | CRPSE | 14 | 37 | 2.24 | | 0.22 | 1 | 8 | 0.16 | 0.05 |
|  |  |  |  | VRE | 9 | 66 | 1.44 | | 0.40 | 7 | 8 | 1.12 | 0.05 |
|  |  |  |  | MRSA | 2 | 39 | 0.32 | | 0.24 | 1 | 14 | 0.16 | 0.08 |
|  | AHEPA University Hospital | 7,287 | 108,176 | CRE | 42 | 76 | 5.76 | | 0.70 | 24 | 41 | 3.29 | 0.38 |
|  |  |  |  | CRAB | 107 | 123 | 14.68 | | 1.14 | 47 | 44 | 6.45 | 0.41 |
|  |  |  |  | CRPSE | 15 | 24 | 2.06 | | 0.22 | 5 | 13 | 0.69 | 0.12 |
|  |  |  |  | VRE | 8 | 31 | 1.10 | | 0.29 | 2 | 18 | 0.27 | 0.17 |
|  |  |  |  | MRSA | 1 | 18 | 0.14 | | 0.17 | 1 | 8 | 0.14 | 0.07 |
|  | Tzaneio General Hospital | 4,613 | 70,727 | CRE | 63 | 27 | 13.66 | | 0.38 | 38 | 14 | 8.24 | 0.20 |
|  |  |  |  | CRAB | 57 | 21 | 12.36 | | 0.30 | 39 | 8 | 8.45 | 0.11 |
|  |  |  |  | CRPSE | 9 | 4 | 1.95 | | 0.06 | 4 | 0 | 0.87 | 0.00 |
|  |  |  |  | VRE | 11 | 21 | 2.38 | | 0.30 | 8 | 2 | 1.73 | 0.03 |
|  |  |  |  | MRSA | 1 | 17 | 0.22 | | 0.24 | 0 | 11 | 0.00 | 0.16 |
| 2021 | Attikon University Hospital | 11,658 | 177,384 | CRE | 128 | 110 | 10.98 | | 0.62 | 55 | 32 | 4.72 | 0.18 |
|  |  |  |  | CRAB | 284 | 141 | 24.36 | | 0.79 | 132 | 59 | 11.32 | 0.33 |
|  |  |  |  | CRPSE | 22 | 27 | 1.89 | | 0.15 | 0 | 7 | 0.00 | 0.04 |
|  |  |  |  | VRE | 16 | 55 | 1.37 | | 0.31 | 14 | 12 | 1.20 | 0.07 |
|  |  |  |  | MRSA | 7 | 23 | 0.60 | | 0.13 | 3 | 10 | 0.26 | 0.06 |
|  | AHEPA University Hospital | 10,443 | 118,468 | CRE | 113 | 127 | 10.82 | | 1.07 | 48 | 38 | 4.60 | 0.32 |
|  |  |  |  | CRAB | 246 | 158 | 23.56 | | 1.33 | 100 | 39 | 9.58 | 0.33 |
|  |  |  |  | CRPSE | 50 | 50 | 4.79 | | 0.42 | 15 | 11 | 1.44 | 0.09 |
|  |  |  |  | VRE | 36 | 87 | 3.45 | | 0.73 | 15 | 37 | 1.44 | 0.31 |
|  |  |  |  | MRSA | 1 | 22 | 0.10 | | 0.19 | 0 | 15 | 0.00 | 0.13 |
|  | Tzaneio General Hospital | 5388 | 87,281 | CRE | 65 | 69 | 12.06 | | 0.79 | 36 | 22 | 6.68 | 0.25 |
|  |  |  |  | CRAB | 83 | 59 | 15.40 | | 0.68 | 50 | 31 | 9.28 | 0.36 |
|  |  |  |  | CRPSE | 7 | 11 | 1.30 | | 0.13 | 7 | 4 | 1.30 | 0.05 |
|  |  |  |  | VRE | 14 | 29 | 2.60 | | 0.33 | 14 | 13 | 2.60 | 0.15 |
|  |  |  |  | MRSA | 2 | 8 | 0.37 | | 0.09 | 0 | 7 | 0.00 | 0.08 |
| 2022 | Attikon University Hospital | 12,992 | 198,608 | CRE | 102 | 148 | 7.85 | | 0.75 | 53 | 49 | 4.08 | 0.25 |
|  |  |  |  | CRAB | 205 | 174 | 15.78 | | 0.88 | 76 | 70 | 5.85 | 0.35 |
|  |  |  |  | CRPSE | 25 | 47 | 2.16 | | 0.24 | 4 | 6 | 0.31 | 0.03 |
|  |  |  |  | VRE | 18 | 86 | 1.39 | | 0.43 | 13 | 20 | 1.00 | 0.10 |
|  |  |  |  | MRSA | 1 | 32 | 0.08 | | 0.16 | 1 | 19 | 0.08 | 0.10 |
|  | AHEPA University Hospital | 10,443 | 121,926 | CRE | 80 | 121 | 7.66 | | 0.99 | 25 | 50 | 2.39 | 0.41 |
|  |  |  |  | CRAB | 105 | 174 | 10.05 | | 1.43 | 43 | 54 | 4.12 | 0.44 |
|  |  |  |  | CRPSE | 29 | 64 | 2.78 | | 0.52 | 5 | 31 | 0.48 | 0.25 |
|  |  |  |  | VRE | 14 | 91 | 1.34 | | 0.75 | 4 | 27 | 0.38 | 0.22 |
|  |  |  |  | MRSA | 4 | 26 | 0.38 | | 0.21 | 2 | 13 | 0.19 | 0.11 |
|  | Tzaneio General Hospital | 5,574 | 83,363 | CRE | 59 | 68 | 10.58 | | 0.82 | 25 | 16 | 4.49 | 0.19 |
|  |  |  |  | CRAB | 66 | 85 | 11.84 | | 1.02 | 31 | 41 | 5.56 | 0.49 |
|  |  |  |  | CRPSE | 29 | 30 | 5.20 | | 0.36 | 9 | 13 | 1.61 | 0.16 |
|  |  |  |  | VRE | 18 | 48 | 3.23 | | 0.58 | 13 | 16 | 2.33 | 0.19 |
|  |  |  |  | MRSA | 4 | 20 | 0.72 | | 0.24 | 1 | 10 | 0.18 | 0.12 |
| Notes: CRAB: carbapenem-resistant *Acinetobacter baumannii*; CRE: carbapenem-resistant *Enterobacterales*; CRPA: carbapenem-resistant *Pseudomonas aeruginosa*; HO: Hospital onset; ICU: Internal care unit; IDR: Incidence Density Rate; LabID: Laboratory-Identified; MRSA: methicillin-resistant *Staphylococcus aureus*; VRE: vancomycin-resistant enterococci | | | | | | | | | | | | | |
