# Supplementary material for: Multidrug-resistant organism bloodstream infection and hospital acquisition among inpatients in three tertiary Greek hospitals during the COVID-19 era
Source: Eur J Clin Microbiol Infect Dis. 2024 Mar 26;43(6):1241–6. doi: 10.1007/s10096-024-04806-x (PMC11178613; doi:10.1007/s10096-024-04806-x)
Supplement: Supplementary file 3 — Supplementary Material 3 [file 10096_2024_4806_MOESM3_ESM.docx]

**Supplementary Figure 1: Monthly pooled carbapenem-resistant Enterobacterales and carbapenem-resistant *Acinetobacter baumannii* Bloodstream Infection Incidence Density Rate (/1,000 patient days) per location in three tertiary Greek hospitals, 2019-2022**

| 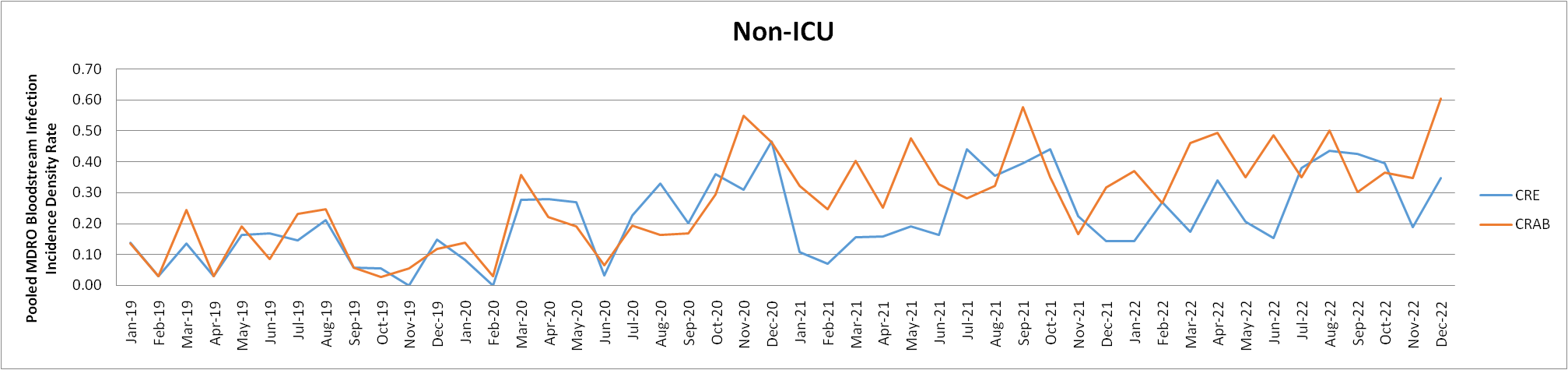 |
| --- |
| 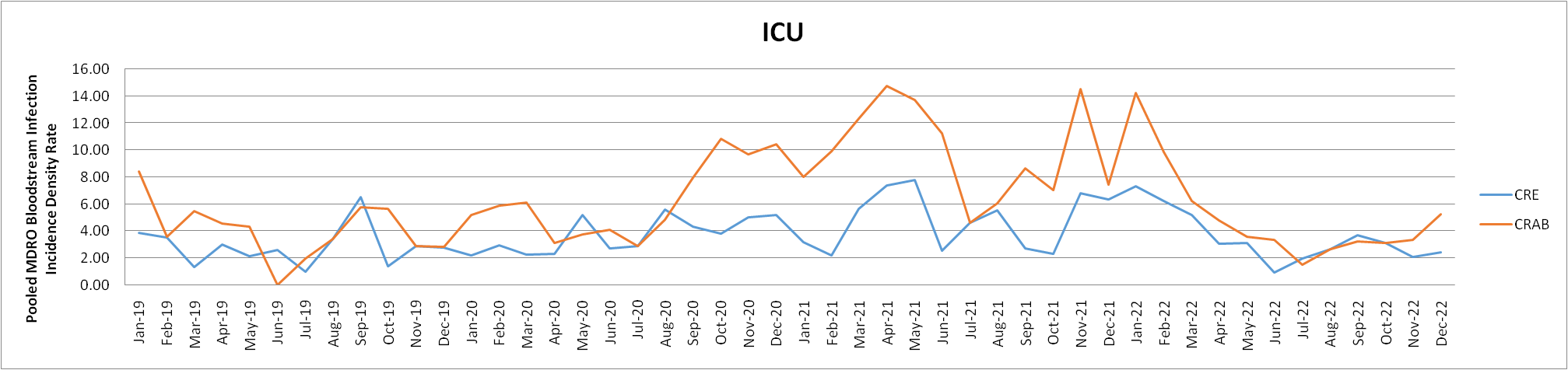 |

Notes: CRAB: carbapenem-resistant *Acinetobacter baumannii*; CRE: carbapenem-resistant Enterobacterales*;* ICU: internal care unit
